# Outcomes and Toxicities of Definitive Radiation Therapy for Unresected Thymic Malignancies

**DOI:** 10.1016/j.jtocrr.2026.100970

**Published:** 2026-02-11

**Authors:** Sana Raoof, Nicolas Toumbacaris, Annemarie F. Shepherd, Daphna Y. Gelblum, Charles B. Simone, Abraham J. Wu, Ellen D. Yorke, Michelle S. Ginsberg, Andrew M. Pagano, James Huang, Gregory J. Riely, Zhigang Zhang, Andreas Rimner

**Affiliations:** a Department of Radiation Oncology, Memorial Sloan Kettering Cancer Center, New York, New York; b Department of Epidemiology and Biostatistics, Memorial Sloan Kettering Cancer Center, New York, New York; c Division of Radiation Oncology, University of Washington School of Medicine, Seattle, Washington; d New York Proton Center, New York, New York; e Department of Medical Physics, Memorial Sloan Kettering Cancer Center, New York, New York; f Department of Radiology, Memorial Sloan Kettering Cancer Center, New York, New York; g Department of Surgery, Thoracic Surgery Service, Memorial Sloan Kettering Cancer Center, New York, New York; h Department of Medicine, Thoracic Oncology Service, Memorial Sloan Kettering Cancer Center, New York, New York; i Department of Radiation Oncology, Medical Center – University of Freiburg, Freiburg im Breisgau, Germany

**Keywords:** Definitive radiation, Thymic malignancies, Thymoma, Thymic carcinoma

## Abstract

**Introduction:**

Thymic malignancies are primarily managed with surgery. Some patients are medically inoperable or technically unresectable. We report the characteristics and outcomes of patients with unresected thymic tumors treated with definitive radiation therapy (RT) at our institution.

**Methods:**

Patients treated with curative-intent RT for unresected thymic tumors between 1997 and 2023 were identified. Age at diagnosis, sex, histology, Masaoka or TNM stage, WHO classification, reason for no resection, RT dose and fractionation, presence of paraneoplastic syndrome, chemotherapy use, and toxicities were extracted. Overall and local failure-free survival, patterns of progression, and treatment toxicities were assessed. This retrospective chart review did not require informed consent.

**Results:**

A total of 33 consecutive patients were identified (16 females and 17 males) with a median age of 61 years at diagnosis. Overall, 22 patients had thymoma and 11 had thymic carcinoma. T stage was T2 (one), T3 (seven), and T4 (25); N stage was N0 (15), N1 (one), and N2 (17); M stage was M0 (21), M1a (eight), and M1b (four). Median tumor size was 8.1 cm. Three patients were medically inoperable; 30 were technically unresectable. Of the 33 patients, 29 received chemotherapy. The median RT dose was 60 Gy in 2 Gy fractions.

Furthermore, 6% developed a grade 3 toxicity and 9% developed grade 2 pneumonitis. No grade 4/5 toxicities were observed. The 2-year overall survival was 69% (95% confidence interval [CI]: 52–92) and 5-year overall survival was 56% (95% CI: 37–85). The 3- and 5-year local failure rates were 20% (95% CI: 6.9–39).

**Conclusions:**

In the most advanced stage and one of the largest reports to date, definitive chemo-RT provides high rates of local control in patients with unresected thymic malignancies.

## Introduction

Thymic malignancies are primarily managed with surgery with or without chemotherapy and radiation therapy (RT). Complete resection is an independent prognostic factor in thymomas.^1^^,^^2^ RT plays a role in the neoadjuvant or more frequently adjuvant settings after complete or incomplete resections. We previously demonstrated that postoperative RT is associated with improved overall survival (OS) in patients with historically staged completely resected Masaoka stage II and III thymomas and locally advanced thymic carcinomas.^3^, ^4^, ^5^ A few patients are medically inoperable (because of age, general frailty, cardiac or pulmonary function, vascular disease, or other comorbidities) or technically unresectable (invasion of the aorta, great vessels, main pulmonary artery, myocardium, trachea, or esophagus).^6^ As per current National Comprehensive Cancer Network guidelines, these patients may be treated with definitive RT with or without concurrent chemotherapy.^7^ There is limited literature on outcomes and toxicities of definitive radiation in these patients. Based on this evidence gap, we analyzed and reported the characteristics and outcomes of patients with unresected thymic tumors who were treated with curative-intent RT at our institution.

## Methods

We conducted a retrospective chart review of all patients treated with curative-intent RT (≥4500 cGy) using conventional fractionation for unresected thymic tumors between 1997 and 2023. We did not include patients treated with definitive salvage RT or stereotactic body RT for local recurrences. Age at diagnosis, sex, histology, TNM stage, Masaoka stage, WHO classification, presence of paraneoplastic syndrome, reason for unresectable status, RT dose and fractionation, use of chemotherapy, survival status, time at local, locoregional, or distant failure, chemotherapy and radiation-related toxicities were extracted.

Operability and resectability were assessed by a dedicated board-certified thoracic surgeon. Treatment toxicities were assessed per Common Terminology Criteria for Adverse Events version 5.0.

The primary outcomes of interest were OS and progression-free survival (PFS). OS was defined as the time from pathologic diagnosis to date of death. PFS was defined from time of pathologic diagnosis to date of local failure, locoregional failure, distant metastases, or death. Patients were censored at the date of last follow-up. The secondary outcome of interest was local failure. Patient characteristics were summarized using count and percentage for categorical variables and median and interquartile range for continuous variables. OS and PFS were analyzed using the Kaplan-Meier method. Local failure was analyzed using cumulative incidence curves. Univariable Fine-Gray competing risk regression was used to analyze associations of clinically relevant factors and local failure. Death without local failure was the competing event. All statistical tests were two-sided with *p* less than 0.05 considered significant. Statistical analyses were conducted using R (version 4.3.3, R Core Development Team, Vienna, Austria).

## Results

### Patient Characteristics

A total of 33 consecutive patients who met the inclusion criteria were included in this study. The median follow-up time was 1.92 years (95% confidence interval [CI]: 1.08–not applicable). Patient and tumor characteristics are found in Table 1. Of the patients, 52% were male and 48% were female. Median age was 61 (interquartile range: 54–69) years. Furthermore, 67% of patients had thymoma compared with 33% with thymic carcinoma. T stage was T2 (one), T3 (seven), and T4 (25); N stage was N0 (15), N1 (one), and N2 (17); T stage was M0 (21), M1a (eight), and M1b (four). Masaoka stage was III in 42%, IVA in 28%, and IVB in 30%; all stage IVB patients had disease confined to the thorax. Paraneoplastic syndrome was present in 15% of patients.

**Table 1:** Characteristics of Patients Included in This Analysis, Including Patient Demographics and Tumor Histology, Stage, and Paraneoplastic Syndrome

Characteristic	n	%
Sex		
Male	17	52%
Female	16	48%
Age at diagnosis	61 (range 28–88) y	
Histology		
Thymoma	22	67%
Thymic carcinoma	11	33%
WHO classification		
A	1	3%
B1	2	6%
B2	12	36%
B3	5	15%
Thymoma not otherwise specified	2	6%
Thymic carcinoma	11	33%
Masaoka stage		
III (42%, n = 14)	14	42%
IVA (28%, n = 9)	9	28%
IVB (30%, n = 10)	10	30%
TNM stage		
T2	1	3%
T3	7	21%
T4	25	76%
N0	15	45%
N1	1	3%
N2	17	52%
M0	21	64%
M1a	8	24%
M1b	4	12%
Median tumor size	8.1 (range 2.2–16.5) cm	
Paraneoplastic syndrome		
Yes	5	15%
No	28	85%

Furthermore, 30 of the 33 patients were considered technically unresectable. In addition, 15 patients were surgically explored and intra-operatively found to be unresectable (common reasons being dense fibrosis precluding planned resection because of lack of surgical planes, mass fixed to the chest wall without feasible complete resection, or mass inseparable from the lung, myocardium, esophagus, trachea or great vessels). The other 15 patients were deemed unresectable based on imaging and clinical information without requiring operation (common reasons being phrenic nerve involvement, invasion of the lung, myocardium, esophagus, trachea or great vessels, pulmonary parenchymal metastases, and other reasons a complete resection was not likely). Three of 33 patients were deemed medically inoperable (one patient had severe diabetic complications and was status post lower extremity amputations and had severe iron deficiency anemia, one was 79 years old and could only walk one block because of shortness of breath at baseline, and one was 87 years old with poorly controlled diabetes).

### Treatment Characteristics

Treatments received are summarized in Table 2. One of 33 patients underwent debulking surgery.

**Table 2:** Characteristics of Treatments Received, Including Surgery, Chemotherapy, and Radiation

Characteristic	n	%
Debulking surgery	1	3%
Chemotherapy		
Yes	29	88%
No	4	12%
Sequential	23	79%
Concurrent	6	21%
Common chemotherapy regimens		
Carboplatin/paclitaxel	7	24%
Cisplatin, doxorubicin and cyclophosphamide	13	45%
Radiation		15%
Median RT dose	6000 (4500–7000) cGy	
Number treated <5400 cGy	5	
Dose per fraction	200 cGy	
Technique		
2D/3D	4	12%
IMRT	24	73%
Protons	5	15%

2D, 2-dimension; 3D, 3-dimension; IMRT, intensity-modulated radiation therapy; RT, radiation therapy.

Furthermore, 29 patients received chemotherapy, and the most common regimen was sequential cisplatin, doxorubicin and cyclophosphamide before RT. Of the 29 patients who received chemotherapy, 23 received it sequentially, and six received it concurrently with RT.

The median RT dose was 6000 cGy in 30 fractions, and most patients included in this analysis (29/33) were treated with the modern techniques of intensity-modulated RT or protons.

### Outcomes

**Figure 1:**
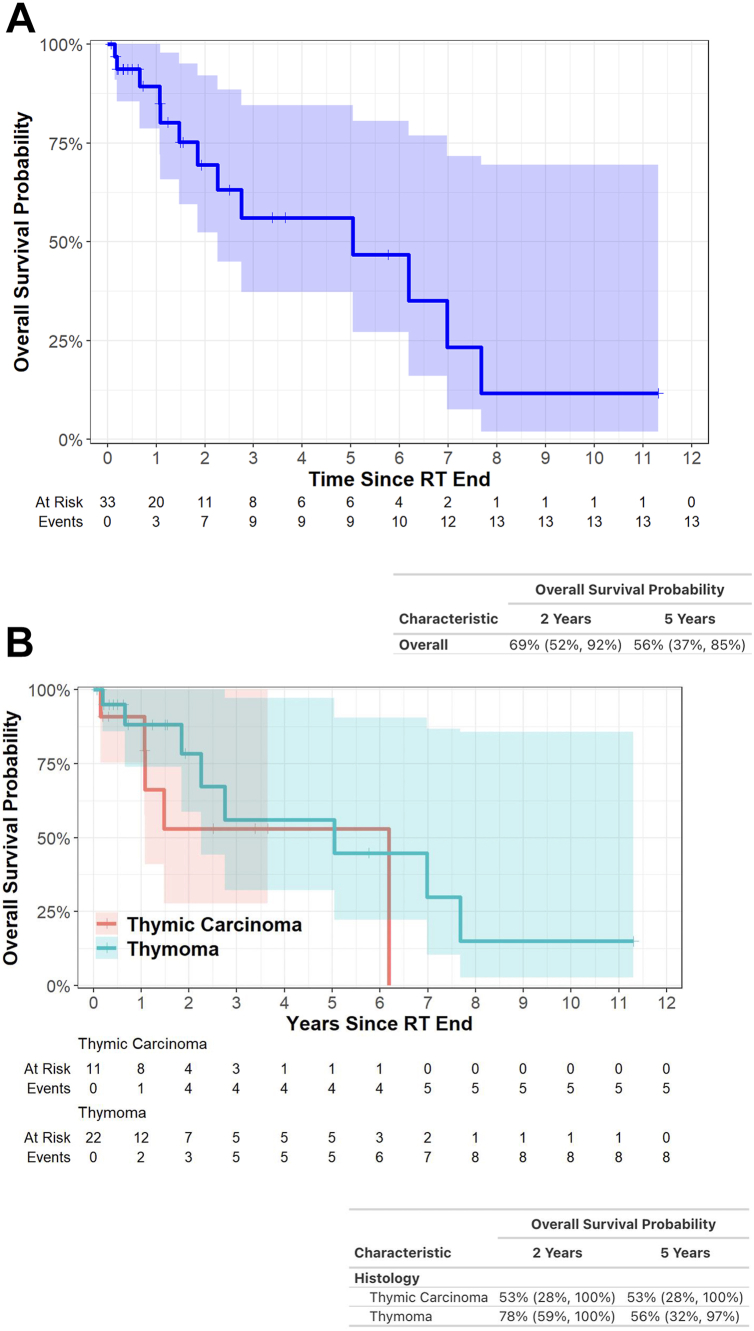
Kaplan-Meier curve illustrating overall survival after definitive radiation for (*A*) thymic malignancies overall and (*B*) thymic malignancies stratified by histology. RT, radiation therapy.

**Figure 2:**
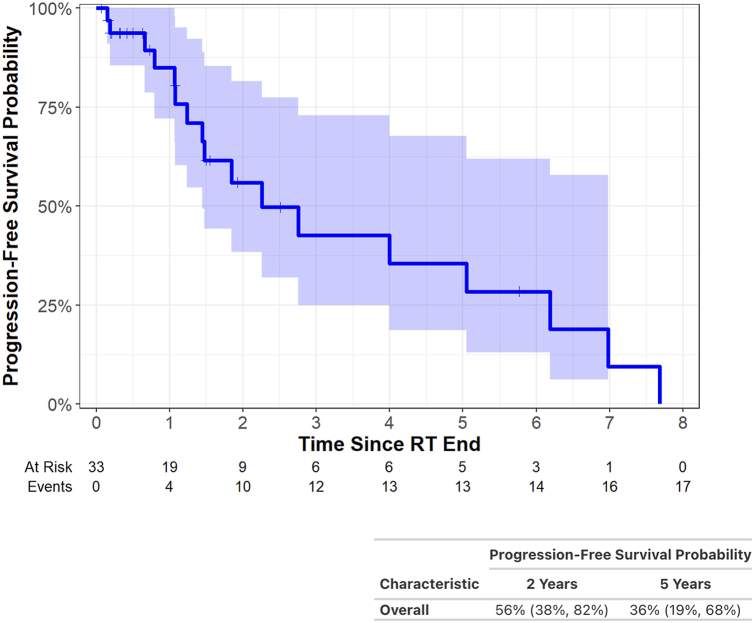
Kaplan-Meier curve illustrating progression-free survival after definitive radiation for thymic malignancies. RT, radiation therapy.

OS at 2 years was 69% (95% CI: 52–92) and at 5 years was 56% (95% CI: 37–85) (Fig. 1*A*). Differences in OS between thymoma and thymic carcinoma patients were not statistically significant (Fig. 1*B*). PFS at 2 and 5 years was 56% (95% CI: 38–82) and 36% (95% CI: 19–68), respectively (Fig. 2). Site of first progression was local in six, locoregional in eight (deep intrathoracic or cervical nodes), and distant in nine (brain, bones, liver, abdominal nodes); several patients progressed in multiple places synchronously.

**Figure 3:**
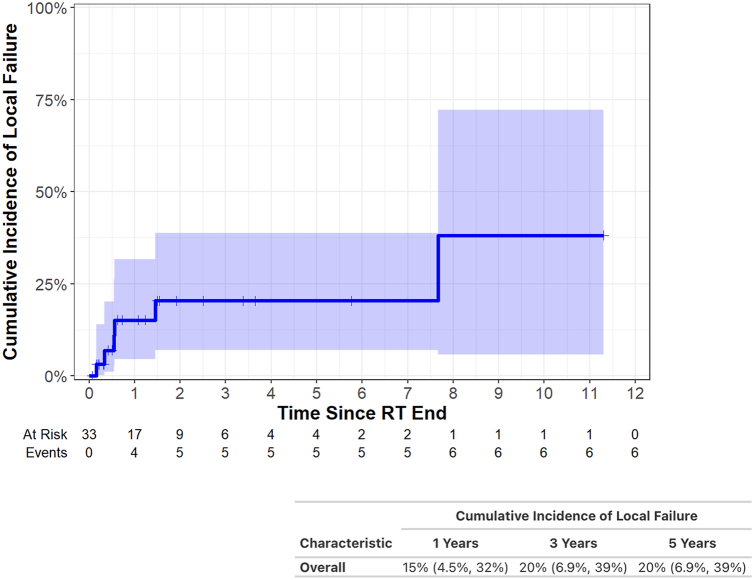
Cumulative incidence of local failure after definitive radiation for thymic malignancies. RT, radiation therapy.

There was a high rate of local control. At 3 and 5 years, the cumulative incidence of local failure was 20% (95% CI: 6.9–39) (Fig. 3), including three patients with thymoma and three patients with thymic carcinoma.

Across all patients, nine experienced distant metastasis by the last follow-up, including four patients with thymoma and five patients with thymic carcinoma. Of 33 total patients, 20 were alive at the last follow-up (Table 3). Of the 13 patients who died, five died of unrelated causes (e.g., preexisting heart disease) and eight died of thymic malignancy, of which five had distant failures, two had locoregional failures, and one had not progressed by that time point.

**Table 3:** Vital Status at Time of Last Follow-Up, Including Cause of Death Among Patients Included in This Case Series

Vital Status at Time of Last Follow-Up	n	%
Alive	20	64%
Died	13	36%
Died of thymic malignancy	8	21%
Local	0	0%
Regional	2	6%
distant	5	15%
Died of complications	0	3%
Died of unrelated causes	5	12%

*Note*: For patients with multiple simultaneous sites of failure (e.g., distant and regional failures), the most advanced type of failure is counted. One patient died of medical complications secondary to thymic malignancy but had neither local, regional, nor distant failure.

Most patients tolerated RT well with one case of grade 3 esophagitis, one case of grade 3 pneumonitis, five cases of grade 2 pneumonitis, one grade 2 fatigue, one grade 2 dermatitis, and one grade 2 dysphagia. No acute or late grade 4 or 5 toxicities were observed. Common and expected grade 1 toxicities included dermatitis, dysphagia, dyspnea, cough, and fatigue.

## Discussion

Patients with thymoma or thymic carcinoma who are surgically resectable should be treated with surgery—only 1.1% of these patients do not receive any form of surgical treatment.^8^ Among patients who are technically unresectable or medical inoperable, definitive radiation is recommended. There is limited literature on outcomes after definitive radiation for thymic malignancies.

One retrospective review of 82 patients with stage III thymoma compared patients treated with RT to at least 54 Gy (n = 54) with patients who were not treated with RT (n = 28). In this cohort, RT was associated with improved OS, PFS, and freedom from locoregional failure, with a 5-year OS of 65.7% and 5-year PFS of 46.1%.^9^ Another retrospective review analyzed 27 patients with stage III or IVA thymoma or thymic carcinoma treated with various nonsurgical modalities, finding that RT to at least 44 Gy improved OS.^10^ In a third series, among 16 patients with unresectable thymic carcinoma, concurrent chemotherapy and RT had an overall response rate of 50% and a complete response rate of 25% at the median follow-up time of 64 months.^11^

We analyzed 33 patients treated with curative-intent RT for unresected thymic tumors between 1997 and 2023 at our institution. This work builds on the existing literature by including patients with late-stage thymic malignancies. Furthermore, all patients were evaluated by a surgeon to be deemed unresectable. Finally, most patients were treated with modern radiation techniques and to doses of at least 5400 cGy, in accordance with Annual Meeting of the International Thymic Malignancies Interest Group (ITMIG) guidelines for definitive RT.^12^ We found that definitive chemoradiation provides high rates of local control in patients with unresected thymic malignancies even in advanced stages. Two-year OS was 69% (95% CI: 52–92) and 5-year OS was 56% (95% CI: 37–85). The 3- and 5-year local failure rates were both 20%. These outcomes are comparable with prior published retrospective reviews mentioned previously. To our knowledge, this analysis represents the second largest case series on outcomes after definitive RT for thymic malignancies and includes patients at later stages than prior reports. We did not have a large enough sample size to stratify results by histology, although previous publications give a clear association of poorer outcomes, including higher risk of relapse and lower OS, with thymic carcinomas after surgery or RT.^13^

In terms of toxicities, we found in our cohort that definitive RT was well tolerated, with only 6% of patients experiencing grade 3 toxicity and no grade 4 or 5 toxicities.

The limitations of this analysis are that it is a retrospective, nonrandomized review. Our analysis is subject to biases, including which patients are deemed inoperable—although this bias was mitigated by only including patients who were assessed, evaluated, and deemed unresectable or inoperable by an experienced thoracic surgeon. Also, we included 15 patients who were surgically explored and found to be unresectable intraoperatively. Furthermore, a variety of RT modalities were included in the current analysis, which can affect the expected toxicity rates reported.

In addition to definitive treatment of unresectable disease, postoperative RT plays a role in improving survival outcomes in completely resected stage II to IV thymomas^3^ and completely resected stage III to IV (locally advanced) thymic carcinomas, including R1-resected thymic tumors.^4^ More research is needed to determine resectability in a noninvasive manner,^14^ optimal sequencing of definitive RT with chemotherapy, optimal RT doses, and best use of modern RT techniques to minimize toxicities.

## CRediT Authorship Contribution Statement

**Sana Raoof**: Investigation, Data curation, Formal analysis, Writing - original draft, Visualization.

**Nicolas Toumbacaris**: Formal analysis, Writing - review & editing.

**Annemarie F. Shepherd**: Data curation, Writing - review & editing.

**Daphna Y. Gelblum**: Data curation, Writing - review & editing.

**Charles B. Simone, II**: Data curation, Writing - review & editing.

**Abraham J. Wu**: Data curation, Writing - review & editing.

**Ellen D. Yorke**: Writing - review & editing.

**Michelle S. Ginsberg**: Writing - review & editing.

**Andrew M. Pagano**: Writing - review & editing.

**James Huang**: Data curation, Writing - review & editing.

**Gregory J. Riely**: Writing - review & editing.

**Zhigang Zhang**: Formal analysis, Writing - review & editing.

**Andreas Rimner**: Conceptualization, Writing - review & editing, Supervision.

## Disclosure

Dr. Riely reports receiving institutional research support from Amgen, Mirati, Lilly, Takeda, Merck, Roche, Pfizer, and Novartis and serving as an uncompensated consultant for Lilly, Takeda, Merck, Roche, and Mirati; none of these disclosures are related to the submitted manuscript. Dr. Rimner reports receiving institutional support from AstraZeneca, Merck, Boehringer Ingelheim, Pfizer, Varian, and Medical System; receiving consulting fees from AstraZeneca, Merck, and More Health; receiving honoraria from Boehringer Ingelheim; receiving travel support from AstraZeneca; having participation in a DSMB for Merck; and having a leadership role in ITMIG (VP), IMIG (Board of Directors), and ABR (Oral board examiner). Dr. Yorke reports receiving grants from NIH P30 CA0087 48. Dr. Wu reports receiving grants from Civa Oncology, Inc., and research grant paid to institution. Dr. Raoof reports serving in the consulting for Grail, Exact Sciences, and Verily; receiving honoraria from Congressional budget office, Dartmouth Grand Rounds, and MGH cancer equity symposium. Dr. Simone reports having a leadership role in the American Society for Radiation Oncology Chair of the Lung Resource Panel and Chair of the Blue Ribbon Lung Panel, Proton Collaborative Group Chair of the Lung Committee, American Radium Society Chair of Thoracic Use Criteria committee, and Particle Therapy Co-Operative Group Chair of the Thoracic Subcommittee. The remaining authors declare no conflict of interest.
